# Correction to “Cell‐engineered virus‐mimetic nanovesicles for vaccination against enveloped viruses”

**DOI:** 10.1002/jev2.12452

**Published:** 2024-05-17

Han, C., Kim, S., Seo, Y., Lim, M., Kwon, Y., Yi, J., Oh, S.‐I., Kang, M., Jeon, S. G., & Park, J. (2024). Cell‐engineered virus‐mimetic nanovesicles for vaccination against enveloped viruses. Journal of Extracellular Vesicles, 13, e12438. https://doi.org/10.1002/jev2.12438

In the originally published article, the acknowledgements section was incorrect. The correct text is as follows:

